# Minocycline treatment suppresses juvenile development and growth by attenuating insulin/TOR signaling in *Drosophila* animal model

**DOI:** 10.1038/srep44724

**Published:** 2017-03-20

**Authors:** Hyun Myoung Yun, Sujin Noh, Seogang Hyun

**Affiliations:** 1 Department of Life Science, Chung-Ang University, Seoul 156-756, Korea

## Abstract

Minocycline is a broad spectrum, semi-synthetic tetracycline analog that is used to treat bacterial infection. Recently, this drug has been receiving increasing attention for its non-antibiotic properties, including anti-inflammatory, tumor suppressive, and neuroprotective effects. *Drosophila* is a useful model organism for studying human metabolism and disease. In this study, we investigated the effects of minocycline on juvenile development and growth in *Drosophila*. Feeding minocycline to *Drosophila* larvae suppresses larval body growth and delays the timing of pupation in a dose-dependent manner. We found that the drug treatment decreased the activated form of Akt and S6K in peripheral tissues, which suggested that the insulin/target of rapamycin (TOR) signaling had been attenuated. Specifically enhancing TOR activity in the prothoracic gland (PG), the ecdysone-generating organ, attenuated the drug-induced developmental delay, which is consistent with the critical role of PG’s TOR signaling in determining pupation time. Our results reveal previously unrecognized effects of minocycline and offer a new potential therapeutic opportunity for various pathological conditions associated with insulin/TOR signaling.

Minocycline (7-dimethylamino-6-desoxytertracycline) is a FDA approved, inexpensive antibiotic drug belonging to the second-generation tetracycline family. Minocycline is not a naturally occurring antibiotic, but has been manufactured semi-synthetically from natural tetracycline antibiotics since 1966. It has been in use for over 30 years against both Gram-positive and Gram-negative bacteria, and is currently prescribed for various symptoms related to bacterial infections, such as acne vulgaris and some sexually transmitted diseases^1^,^2^. Minocycline shows a superior pharmacokinetic profile than its original tetracycline parent when administered orally. It is rapidly and completely absorbed in the human body, has a longer half-life in the serum, and shows good tissue penetration^3^. Furthermore, it is known to be safe when used chronically, and is lipophilic enough to go through the blood brain barrier^2^,^4^,^5^.

Numerous studies have recently highlighted the non-antibiotic properties of minocycline, such as anti-inflammatory and anti-apoptotic activities, as well as suppressive effects on cancer metastasis and angiogenesis^6^,^7^,^8^,^9^. These characteristics support the potential use of the drug in the treatment of diseases, such as rheumatoid arthritis, inflammatory bowel syndromes, scleroderma, aortic aneurysms, and malignant tumors^6^,^10^,^11^. Furthermore, based on its ability to cross the blood brain barrier to reach the central nervous system, minocycline has also emerged as a candidate therapeutic agent for the treatment of various neurological diseases. Several studies have demonstrated that minocycline inhibits neuroinflammation and neuron death in animal models of Alzheimer’s disease, amyotrophic lateral sclerosis, Huntington’s disease, Parkinson’s disease, and strokes^12^,^13^,^14^,^15^,^16^,^17^.

Although the mechanism of action behind the antibiotic properties of tetracycline derivatives, including minocycline is well understood, the exact mechanisms of action underlying the non-antibiotic properties of minocycline, such as anti-inflammatory, immunomodulatory, and neuroprotective effects, have only just begun to be explored.

*Drosophila* has been a popular eukaryotic organism in heredity and biomedical research. This organism has been used in biological research since the early 1900s, which means that there are a large number of experimental tools that can be used to facilitate genetic studies. Importantly, *Drosophila* has many parallels with humans in several aspects of animal physiology, including the mechanisms underlying nutritional energy metabolism, juvenile body growth, and sexual maturation^18^,^19^,^20^. Both organisms show exponential body growth in the juvenile period, followed by a sexual maturation process that coincides with growth termination. Insulin/insulin like growth factor signaling (IIS) and steroid signaling play a major role during this process, our current understanding of which has hugely benefited from studying the *Drosophila* model organism^18^,^19^.

In this study, we tested the potential effects of minocycline on *Drosophila* development and growth. Feeding minocycline to *Drosophila* larvae extends the duration of the larval period by delaying pupation time, but does not affect survival rate. The overall growth rate of the larvae declines and the final body size of the adult are reduced when it is feeding on minocycline. We found that minocycline treatment suppresses the insulin/target of rapamycin (TOR) signaling in larval tissues, which is shown by decreased levels of the activated forms of phospho-Akt and phospho-S6K. Finally, enhancing TOR signaling in the prothoracic gland (PG), an endocrine organ that produces ecdysone steroid hormone, which dictates initiation of pupation, attenuates developmental delays induced by minocycline. This provides the underlying mechanism for minocycline effects on larval development.

## Results

### Minocycline feeding suppresses ecdysone signaling, thereby delaying larval development

**Figure 1:**
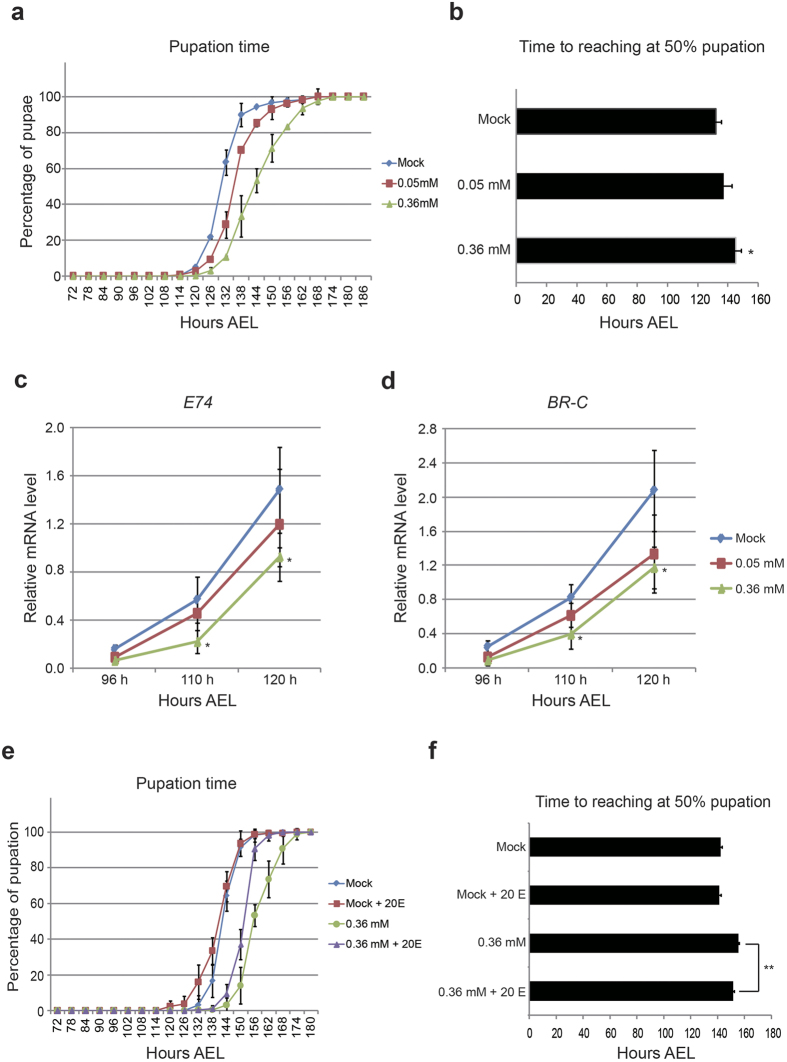
Feeding minocycline to larvae suppresses larval development by delaying pupation time. (**a**) The cumulative percentage of the puparia population formed is shown over time. For determining pupation time, the number of puparia formed was counted every 6 hours AEL (After egg laying) when wild-type larvae (*w*^*1118*^) entered late 3^rd^ larval stage. (**b**) The bar graph shows time when 50% of animals pupated. The pupation time is delayed in a dose-dependent manner when larvae are fed minocycline. Three vials, each containing ~50 larvae were examined per treatment (**a**,**b**). Increases in the mRNA levels of *E74* (FBgn0000567) (**c**) and *BR-C* (FBgn0283451) (**d**), two early response genes of ecdysone signaling, as the larvae develop toward pupation. We isolated total RNA from five mid 3^rd^ instar larvae (96 h AEL) and three late 3^rd^ instar larvae (110 h and 120 h AEL), respectively. Minocycline feeding suppresses the increases in*E74* and *BR-C*. The expression level of *E74* and *BR-C* were normalized by *Rp49* mRNA level. The values were from three independent experiments. (**e**) The pupation time is partially rescued when minocycline-feeding larvae are supplemented with 20E from early 3^rd^ larval stage (72 h AEL). (**f**) The bar graph shows time when 50% of animals pupated. Three vials, each containing 30 larvae, were examined per treatment. The values were from three independent observations, each with 30 larvae. Graphs represent mean ± S.D. *p < 0.05 when compared to the respective controls (t-test).

To examine the effect of minocycline on *Drosophila* larval development, we prepared foods that contained minocycline at low (0.05 mM) and high (0.36 mM) concentrations, collected embryos on these foods, and reared the newly hatched larvae until pupation. We examined whether there were any abnormalities in larval development when the larvae were fed minocycline by examining pupation time and larval size. It was found that larvae fed fly food containing minocycline became pupa at a later time than larvae fed normal fly food containing no minocycline (Fig. 1a,b). Delayed pupation was more pronounced when the concentration of minocycline was higher (Fig. 1a,b). Ecdysone, an insect maturation hormone, plays a critical role in dictating developmental transitions, such as larval molting and pupation. When larvae undergo the late 3^rd^ larval instar stage, the ecdysone level in larval hemolymph dramatically increases, which activates ecdysone receptor (EcR) mediated signaling and subsequently induces pupation. We examined whether the activation of ecdysone signaling was suppressed when minocycline was present. Activation of ecdysone signaling was monitored by examining the changes in the mRNA levels of *E74* and *BR-C*, the early response genes of ecdysone signaling^21^. Indeed, when the larvae approached the onset of pupation, the rate of increase in the mRNA levels of *E74* and *BR-C* in larvae fed minocycline was slower than in the larva fed no minocycline, which indicated that minocycline treatment suppressed ecdysone signaling (Fig. 1c,d). However, when minocycline-feeding larva reached wandering stage, the level of ecdysone signaling became eventually similar to that in wandering control larva (Supplementary Fig. S1). This data points to an underlying molecular pathway for prolonged larval development and delayed pupation. To verify our hypothesis further, we treated 20E (20-hydroxyecdysone), a biologically active form of ecdysone, on minocycline feeding larvae from early 3^rd^ instar larval stage (72 h AEL). The result showed that treating 20E significantly advanced the timing of pupation in minocycline treated larvae, without affecting mock-treated larvae (Fig. 1e,f). It is noted that 20E treatment did not fully rescue the developmental delay, implying that other mechanism than control of ecdysone production might also contribute to delayed larval development induced by minocycline.

Then, we investigated whether minocycline feeding has any deleterious effects on animal survival. The results showed that minocycline treatment neither interfered with the pupal formation or adult eclosion (Table 1), precluding the decline of overall animal fitness induced by the drug, which is consistent with previous observations^22^,^23^. Thus, we can conclude that minocycline suppresses ecdysone signaling, thereby delaying larval development and pupation time.

### Minocycline feeding suppresses larval growth and final adult size

**Figure 2:**
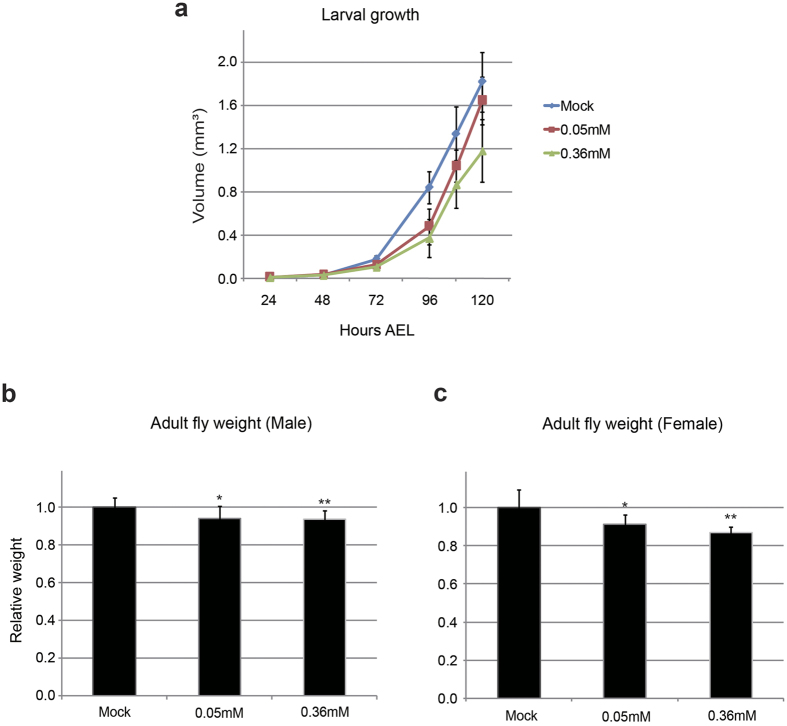
Minocycline feeding suppresses larval body growth and decreases final adult size. (**a**) Larval volumes of *w*^*1118*^ were measured as the larvae developed toward puparium formation. Minocycline feeding suppresses larval body growth in a dose-dependent manner. ~35 wild type larvae from 1^st^ instar larvae (24 h AEL) to late 3^rd^ instar larvae (120 h AEL) in each treatment were measured. (**b**) Final adult masses were measured. Feeding minocycline results in a decrease in the size of the adult flies for both sexes. ~30 flies in each treatment were measured. Graphs represent mean ± S.D. *p < 0.05 **p < 0.01 when compared to the respective controls (t-test).

We investigated whether minocycline feeding has any effects on larval growth. Larval volumes at several time points during development from newly hatched larvae until late 3^rd^ instar larvae were measured. The results showed that minocycline feeding decreased the overall rate of larval body growth and this phenotype became more apparent as the drug concentration increased (Fig. 2a). Next, to further confirm whether minocycline’s effect on larval growth has a sexual bias, we segregated larvae by sex, and measured larval volumes after early 3^rd^ instar larval stage. The results showed that minocycline suppresses larval body growth regardless of sexes (Supplementary Fig. S2a). It has been thought that in metazoans, including *Drosophila*, final adult size is determined during the juvenile growth period and adult size is largely fixed after completion of sexual maturation^18^,^19^. Since larval growth was retarded when *Drosophila* was fed minocycline, we examined whether minocycline feeding during the larval period also affected final adult size. The results showed that minocycline feeding during the larval period significantly reduced the final adult size in a drug dosage dependent manner (Fig. 2b,c and Supplementary Fig. S3). The growth suppressive effect was seen in both male and female flies. It is noted that suppressive effect of minocycline on final adult size was less than its effect on larval growth, which could be explained by compensation of adult size by extended larval growth period. Then, we investigated whether decreased body size was due to reduced cell number or reduced cell size. The examination of adult wing cell number and wing cell size by counting wing bristle indicated that only wing cell number was slightly decreased (Supplementary Fig. S2b,c). Collectively, our data indicate that minocycline influences *Drosophila* larval development by delaying pupation timing and suppressing larval body growth.

### Minocycline feeding attenuates insulin/TOR signaling in larval tissues

**Figure 3:**
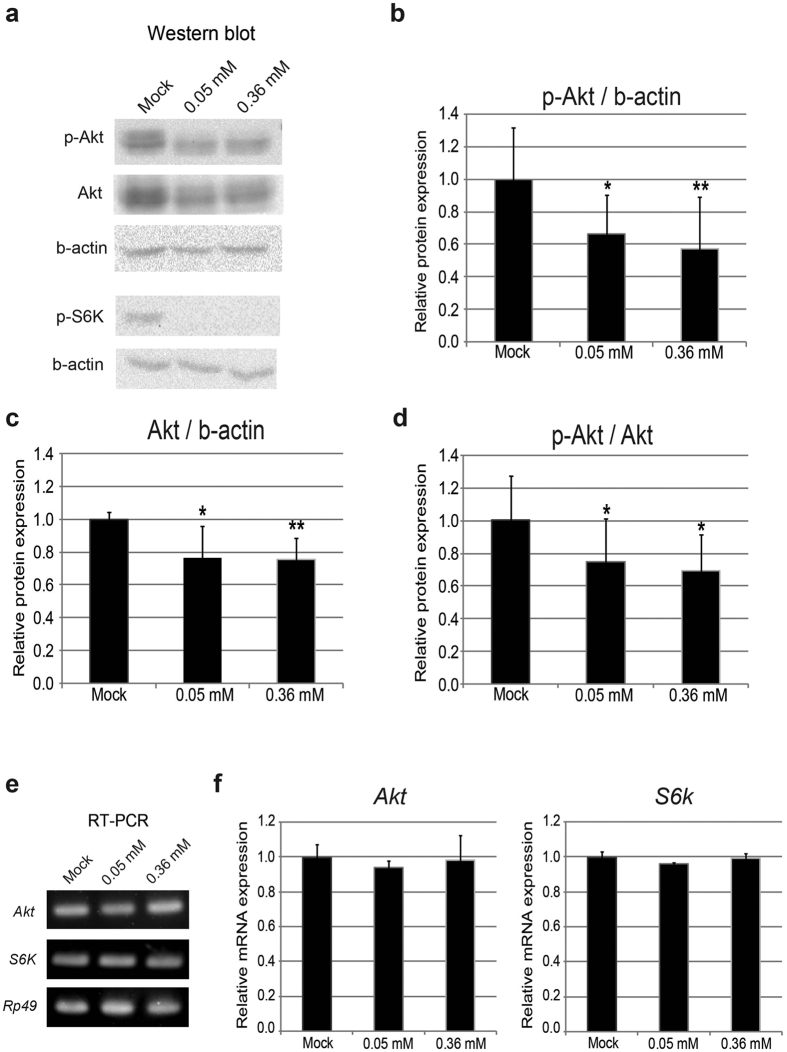
Minocycline feeding suppresses insulin/TOR signaling in larval tissues. (**a**) The amounts of phosopho-Akt (p-Akt), Akt, phospho-S6K (p-S6K), and beta actin (b-actin) were measured by western blot using lysates from early 3^rd^ instar larval bodies (72 h AEL) of *w*^*1118*^. Minocycline treatment significantly decreases the amounts of p-Akt, Akt, and p-S6K. (**b–d**) Quantification of replicated results from the western blot analyses. (**e**) The mRNA levels of *Akt* (FBgn0010379) and *S6K* (FBgn0283472) were measured by RT-PCR. We isolated total RNA from ten early 3^rd^ instar larvae (72 h AEL). Minocycline treatment does not affect the *Akt* and *S6K* mRNA levels. (**f**) Measurement of mRNA levels of *Akt* (FBgn0010379) and *S6K* (FBgn0283472) using qRT-PCR. At least three biological replicates from each of the samples were analyzed and the data are presented as mean ± S.D. *p < 0.05 **p < 0.01 when compared to the respective controls (t-test).

Insulin/TOR signaling is known to play a critical role in regulating larval growth and in determining the pupation timing in *Drosophila*^18^,^19^,^20^. Reduced insulin/TOR signaling induced by poor nutrition in the larvae postpones the timing of pupation and suppresses the rate of larval body growth. Since larvae fed minocycline exhibited retarded growth and delayed pupation, we investigated whether feeding the drug to larvae had any influence on this signaling. Activation of insulin receptors by insulin peptides triggers the cascade activation of downstream effect or proteins by phosphorylation. Akt is the central mediator protein of insulin signaling that is phosphorylated in response to activation of the insulin receptor^18^,^24^. Thus, we examined the activity of insulin signaling in larval tissues by monitoring the insulin-activated form of phospho-Akt by western blot using larval extracts. The results showed that larvae that had been fed minocycline had lower levels of phospho-Akt in whole larval tissues than larvae that were not fed minocycline (Fig. 3a,b). The target of rapamycin (TOR) protein kinase is another branch of the signaling cascade that is closely associated with insulin signaling. TOR can be activated by intracellular amino acids and by activation of Akt during insulin signaling when nutritional conditions are rich^18^,^24^. Minocycline treatment decreased the phospho-S6K, the activated form of S6K induced by TOR activation, in larval tissues (Fig. 3a), which is consistent with Akt repression by minocycline.

Intriguingly, we observed that minocycline treatment not only decreased the amount of phospho-Akt, but also decreased the amount of total Akt (Fig. 3a–c). To investigate whether minocycline affects the level of Akt by influencing the transcription of the *Akt* gene, the mRNA levels for *Akt* were examined by RT-PCR in parallel with Akt protein by western blot analysis. The results showed that there was no change in the mRNA levels of *Akt* during minocycline treatment, which indicated that minocycline affects Akt expression at the post-transcriptional level (Fig. 3e,f). Furthermore, a decrease in the relative amounts of phospho-Akt to total Akt was observed (Fig. 3a,d), which suggests that minocycline also suppresses the activity of insulin signaling upstream of Akt.

To investigate the minocycline effect on insulin signaling further, we examined mRNA levels of insulin signaling components upstream or downstream of Akt in larval tissues. Notably, minocycline significantly repressed the expression of several genes upstream of Akt, including *chico*, an insulin receptor substrate (IRS), and *Dp110*, a homolog of human phosphatidylinositol-4,5-bisphosphate 3-kinase (PI3K) (Supplementary Fig. S4). Unexpectedly, however, expression of Forkhead box O (FOXO), a crucial transcription factor negatively regulated by Akt, as well as its target genes were repressed (Supplementary Fig. S5), implying that minocycline effect on larval development and growth seen in this study appears independent of FOXO (see discussion). Collectively, our data suggest that minocycline suppresses insulin signaling at multiple levels in larval body.

### Enhancing TOR signaling in the prothoracic gland attenuates minocycline-induced delay of larval development

**Figure 4:**
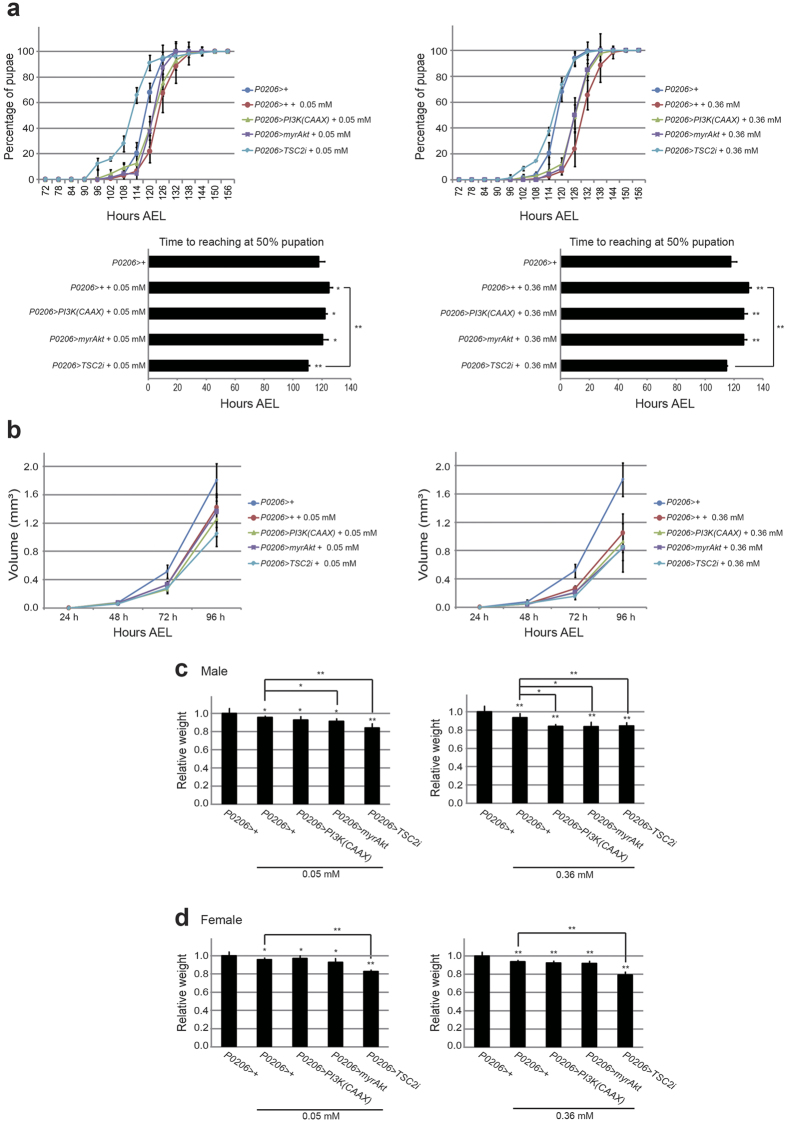
Enhancing TOR signaling in PG attenuates developmental delays caused by minocycline treatment. (**a**) Knockdown of TSC2, the repressor of TOR, specifically in the PG mitigates minocycline-induced delay in pupation time. Overexpression of active form of PI3K [PI3K(CAAX)] or Akt (myrAkt) specifically in the PG fails to rescue minocycline-induced delay in pupation time. Three vials, each containing ~50 larvae were examined per treatment. (**b**) Enhancing insulin/TOR signaling in the PG do not rescue the growth suppression induced by minocycline feeding. ~25 larvae in each treatment were measured. (**c**) Adult male and (**d**) female masses were measured. Enhancing TOR in the PG with minocycline treatment causes significant reduction of final adult size. ~30 flies in each treatment were measured. Graphs represent mean ± S.D. *p < 0.05 **p < 0.01 when compared to the respective controls (t-test).

To further support the idea that minocycline effects on *Drosophila* larval development are caused by a decrease in insulin/TOR signaling, we attempted to rescue the minocycline-induced developmental delay by manipulating insulin/TOR signaling. The prothoracic gland (PG) is the insect endocrine organ that produces ecdysone. Recent studies have indicated that insulin/TOR signaling in PG plays a critical role in determining the pupation time in response to organismal nutritional conditions by regulating ecdysone production^25^,^26^,^27^. As such, decreased insulin/TOR signaling in PG prolongs the larval period by delaying pupation time. Thus, we tested whether genetically enhancing either insulin or TOR signaling specifically in the PG might prevent the effect of minocycline in delaying pupation time. TOR can be activated by suppressing Tuberous Sclerosis complex 2 (TSC2), which is the protein that acts as a TOR repressor. Using the PG specific Gal4 driver (*P0206 Gal4*), RNA interference (RNAi) was induced to knockdown of TSC2. To our expectation, this genetically manipulated *Drosophila* mitigated the minocycline’s effect of pupation time delay (Fig. 4a). This phenotype was consistently seen when different RNAi lines against *TSC2* and different PG specific Gal4 (*phm Gal4*) were used (Supplementary Fig. S6). However, genetically enhancing insulin signaling by expressing either an active form of Akt (a myristoylated Akt; myrAkt) or an active form of PI3K [PI3K(CAAX)] in the PG failed to rescue minocycline-induced developmental delay (Fig. 4a). This result suggested that although minocycline treatment broadly affects insulin/TOR signaling components, suppression of TOR signaling in PG is responsible for minocycline-induced larval developmental delay. It is noted that the PG specific activation of insulin/TOR signaling did not rescue reduced larval growth and final adult size (Fig. 4b–d and Supplementary Fig. S7), which indicated that the growth phenotype is mediated by other organ(s) targeted by minocycline than PG.

## Discussion

Since minocycline was first manufactured in the 1960s, it has long been used for a broad range of symptoms caused by bacterial infections, such as acne vulgaris. Much attention has recently been paid to its further effectiveness in various disease conditions not associated with bacterial infections. Numerous studies have shown that minocycline has the potential to alleviate inflammation-related disorders, to suppress tumorigenesis, and to inhibit angiogenesis^6^,^7^,^8^,^9^,^28^. Notably, minocycline has the ability to readily cross the blood brain barrier, which currently pushes the investigation of minocycline as a potential therapeutic agent for many neurological disorders, such as Alzheimer’s disease, amyotrophic lateral sclerosis, Huntington’s disease, and Parkinson’s disease^13^,^14^,^15^,^16^,^17^. Although minocycline’s curative potential for various medical conditions has been increasingly revealed, the underlying mechanisms for these have only just begun to be understood. In this study, we employed the *Drosophila* model system to investigate the potential effects of minocycline on animal development and their underlying mechanisms. It was found that larvae fed on minocycline developed slowly because pupation time was delayed, and there was a decreased overall rate of body growth resulting in smaller sized adults. Since insulin/TOR signaling in larvae is implicated in the regulation of pupation timing and larval growth, we examined the effects of minocycline on insulin/TOR signaling in larval tissues. Minocycline attenuates insulin/TOR signaling in larval tissues, which is shown by decreased levels of phospho-Akt and phospho-S6K. Interestingly, treatment with minocycline not only decreased the relative amount of phospho-Akt compared to total Akt, but also lowered the level of total Akt, without affecting its mRNA level. Moreover, the drug treatment also repressed the expression of *chico* and *Dp110*, the proteins encoded by which act upstream of Akt in insulin signaling. Thus, minocycline appears to influence the insulin/TOR signaling at multiple different levels. Furthermore, genetically activating TOR signaling in the prothoracic gland (PG), the ecdysone generating organ that is responsible for pupation timing, normalized the pupation time affected by minocycline. This indicated that reduced TOR signaling in PG was a mechanism underlying pupation delay.

Unexpectedly, despite reduced Akt and TOR activity, mRNA expressions of *FOXO* and its target genes, *4E-BP* and *Step*, were reduced upon minocycline treatment (Supplementary Fig. 5). This result implies that minocycline treatment affects IRS-PI3K-Akt-TOR (Fig. 3 and Supplementary Fig. S4) pathway thereby influencing larval development, but this developmental effect is independent of FOXO. It might be postulated that minocycline negatively regulates expression of *FOXO* gene independently of Akt, thereby preventing the upregulation of FOXO by Akt suppression.

There have been several proposed molecular targets of minocycline that have attempted to explain the non-antibiotic properties of the drug. Increased production of reactive oxygen species underlies numerous pathological conditions and leads to poor regulation of cellular homeostasis and damage to the cell^29^. Due to the multi-substituted phenol ring, minocycline has the ability to scavenge free radicals and thereby protects against oxidative stress^30^. It has also been shown that the drug physically interacts with apoptotic protease activator factor-1 (Apaf-1), which inhibits caspase activation and cellular apoptosis^31^. Minocycline is known to inhibit some enzymes, such as metalloproteinases and oxygenases, the generators of reactive oxygen species, thus attenuating inflammatory responses^32^,^33^. Our study further expands the molecular target repertoire of minocycline by showing that the drug downregulates the activities of Akt and S6K, the main effect or components in the insulin/TOR signaling pathway. Our findings may provide a plausible explanation for many of the non-antibiotic minocycline effects. For example, minocycline and its derivatives have been shown to have anti-tumor properties in various cancers, such as leukemia, prostate, and ovarian cancers^34^,^35^,^36^, which could be explained by minocycline’s suppression of insulin/TOR signaling. In line with this, it would be worth noting that rapamycin, a well-known anti-cancer agent that block TOR activity, has similar effect on *Drosophila* larval development and growth as minocycline (Supplementary Fig. S8). Future studies will be necessary to confirm the minocycline effects on insulin/TOR signaling in various organisms including human.

## Methods

### Fly rearing and drug treatment

*w*^*1118*^ was used as a control fly^37^ obtained from Bloomington stock center (BL 3605). *P0206 Gal4* was a gift from Dr. Christan Klämbts. *Phm Gal4* was a gift from Dr. Mike O’Connor. *UAS-PI3K(CAAX*) and *UAS-myrAkt* were gifts from Dr. Jongkyoung Chung. Three different *UAS-TSC2 RNAi* lines were from Vienna (VDRC 103417, VDRC 6313) and Kyoto stock center (NIG 6975R-8). All stocks were maintained at 25 °C with 60% humidity and 12-h light and dark cycles on standard glucose medium: 862 g dextrose anhydrous, 408 g cornmeal, 624 g dried yeast, and 93 g agar, mixed in 10 L water, and boiled. After cooling under 65 °C, 130 ml of 10% methyl 4-hydroxybenzoate stock (H3647, Sigma Aldrich) and 50 ml of propionic acid (79-09-4, Junsei) were added as preservatives. Minocycline stock solution (45 mM) was prepared by dissolving minocycline hydrochloride (M9511, Sigma Aldrich) in distilled water and mixed in medium with appropriate final concentrations. Rapamycin stock solution (100 mM) was prepared by dissolving rapamycin powder (37094, Sigma Aldrich) in DMSO and was stored at −70 °C. Rapamycin stock was mixed in medium under 65 °C with appropriate final concentration.

### Measurements of larval growth, pupation time and adult fly weight

Embryos were collected for 4 hours in normal medium and minocycline-treated medium and reared at the same time. For measurement of larval volume, larvae were treated with boiling water. Digital images of larva were captured by Toupview software (Touptek) and larval volume was measured by using the formula 4/3π(*L*/2)(*l/*2)^2^ (*L* = length *l* = width). For determining pupation time, the number of puparia formed was counted as larva passed by late 3^rd^ larval stage. About 50 larvae were reared in food vials to avoid overcrowding. To measure adult body weight, the groups of 3 virgin flies of both sexes not older than 5 hours after eclosion were weighed. Total 10 groups of flies were weighed for each condition. All flies were ice-anesthetized before weight measurement.

### 20-hydroxyecdysone (20E) treatment

The stage-synchronized early 3^rd^ instar larvae feeding normal medium or minocycline-treated medium were quickly transferred into medium containing appropriate amount of either ethanol or 20E stock (0.45 mg/ml) (H5142, Sigma Aldrich).

### Survival rate

Thirty larvae of 1^st^ larval instar were transferred to minocycline-treated or untreated fly food and were reared until puparitaion. Number of pupa formed was counted and the ratio of the number of pupa to larva was calculated. After eclosion of the adult fly from the pupa, the number of eclosed flies was counted and the ratio of the number of adult flies to pupa was calculated.

### RNA preparation and Quantitative RT-PCR (qRT-PCR)

Whole larval bodies were quickly transferred to Trizol solution (Invitrogen) and grinded for RNA preparation. RNA was extracted following the manufacturer’s recommended instructions. cDNAs were synthesized using RevertAid Reverse Transcriptase (Thermo Scientific). PCR was performed using the CFX Connect Real-Time PCR Detection System (Bio-Rad) and SYBR PCR master mix (TaKaRa). All interested mRNA levels were calculated as a relative fold-change over *Rp49* mRNA. The comparative cycle threshold (Ct) method was applied to estimate mRNA levels. For amplifying *Akt* and *S6K*, PCR was performed for 28 and 22 cycles, respectively, and PCR products were analyzed by agarose gel electrophoresis. The following PCR primers were used: *E74*, 5′-CATCCACGAACTGGTAGAC-3′ and 5′-ACATGAACTACGAGACGATG-3′; *BR-C*, 5′-AGGAGATCGGCGACGGAC-3′ and 5′-TTGAGACCTAGCAACGCTGAG-3′; *Akt*, 5′-CTTTGCGAGTATTAACTGGACAGA-3′ and 5′-GGATGTCACCTGAGGCTTG-3′; *S6K*, 5′-GCTTCCAAGGAGGCTTCCGC-3′ and 5′-TGGACTCGCGAATGGAGGCA-3′; *FOXO*, 5′-GCAATCAACAAGGAGCAACA-3′ and 5′-TCGCCAGCCCAAAAGATA-3′; *4E-BP*, 5′-AGACCAAGTCGCTGAAGATTG-3′ and 5′-TGACCGAGAGAACAAACAAGG-3′; *Step*, 5′-CGCAGTCCATAAGCCATAATC-3′ and 5′-AGCAACACAAGCGAGTAGCA-3′; *InR*, 5′-CCCTGTTCGGCTATGTCTGT-3′ and 5′-AATGATGTTCTCTCGCAGCA-3′; *Chico*, 5′-AGCATTACCAAGGAAGGAAC-3′ and 5′-ATTCGGTCGGAGTTTATCTG-3′; *Dp110*, 5′-CCATCCAGGTAATCAAGGTG-3′ and 5′-GTTTCGTCGGTGTAAGGTTC-3′ *Rp49*, 5′-AGGGTATCGACAACAGAGTG-3′ and 5′-CACCAGGAACTTCTTGAATC-3′.

### Western blot

Whole larval bodies were homogenized in lysis buffer (1% Triton X-100, 50 mM Tris pH 7.4, 500 mM NaCl, 7.5 mM MgCl_2_, 0.2 mM EDTA, 1 mM Na_3_VO_4_, 50 mM NaF, 1 mM DTT, 25% glycerol). The isolated protein samples were quantified by Bradford assay and separated in 10% poly-acrylamide gel. After transfer, Immobilon^TM^ PVDF membrane (Millipore) were blocked for 1 h at room temperature (RT) in TBST containing 5% skim milk and incubated at 4 °C for overnight with anti-Akt (#9272, Cell signaling), anti-phospho-Akt (#4054, Cell signaling), anti-phospho-S6K (#9209, Cell signaling) or anti-β-actin (#4967, Cell signaling) antibodies. All primary antibodies were diluted at 1:1,000 with 5% BSA in TBST. After washing, the membrane was incubated with 5% skim milk in TBST containing HRP-conjugated secondary antibody (1:10,000) at RT for 1 h. HRP signal was generated by ECL substrate treatment and was analyzed with ChemiDoc XRS system (Bio-Rad).

### Statistical analysis

Standard deviation (S.D.) is denoted as error bar for measured values in larval volume, pupation time, adult weight, quantitative RT-PCR, and western blot experiments. P values are calculated by two-tailed Student’s t-test using Excel software (Microsoft) or calculated by log-rank test using OASIS website^38^.

## Additional Information

**How to cite this article:** Yun, H. M. *et al*. Minocycline treatment suppresses juvenile development and growth by attenuating insulin/TOR signaling in *Drosophila* animal model. *Sci. Rep.* **7**, 44724; doi: 10.1038/srep44724 (2017).

**Publisher's note:** Springer Nature remains neutral with regard to jurisdictional claims in published maps and institutional affiliations.

## Supplementary Material

Supplementary Information

## Figures and Tables

**Table 1:** Survival rate of larva until adult eclosion.

Larva to pupa			*^#^Pupa to adult		
Treatment	Mean ± S.D. (%)	P-value	Treatment	Mean ± S.D. (%)	P-value
Mock	81.66 ± 1.44	-	Mock	98.95 ± 1.82	-
0.05 mM	82.50 ± 7.50	0.85	0.05 mM	97.03 ± 2.79	0.37
0.36 mM	80.00 ± 4.33	0.56	0.36 mM	98.92 ± 2.00	0.98

^*^Adult number was counted at 2 days after eclosion. ^#^No sex-biased effects were seen.
