# Supplementary material for: Minocycline treatment suppresses juvenile development and growth by attenuating insulin/TOR signaling in Drosophila animal model
Source: Sci Rep. 2017 Mar 20;7:44724. doi: 10.1038/srep44724 (PMC5357794; doi:10.1038/srep44724)
Supplement: Supplementary Information [file srep44724-s1.pdf]

Minocycline treatment suppresses juvenile development and growth by attenuating insulin/TOR signaling in *Drosophila* animal model

Hyun Myoung Yun[1], Sujin Noh[1] and Seogang Hyun[1*]

[1]Department of Life Science, Chung-Ang University, Seoul 156-756, Korea

Phone: + 82-2-820-5805
Fax: +82-2-825-5206
*Correspondence: sghyun@cau.ac.kr (S.H.)

Running title: Minocycline effect on animal development

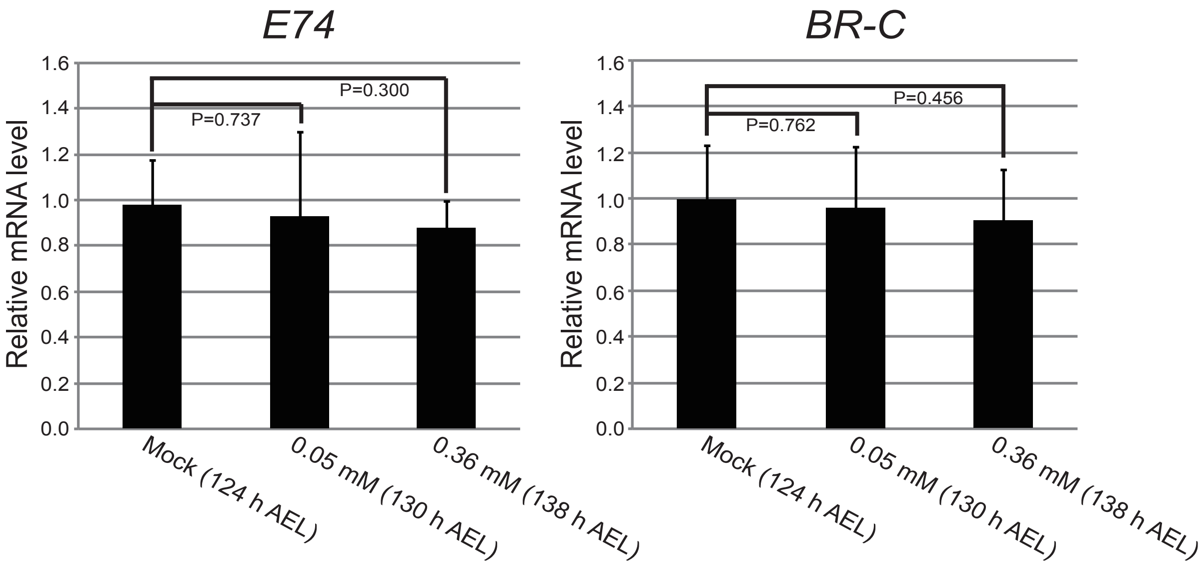

Supplementary figure S1. Similar levels of ecdysone signaling between minocycline feeding larvae and mock treated larvae when measured at wandering stage. Analysis of *E74* (FBgn0000567) and *BR-C* (FBgn0283451) transcripts level in early wandering staged larvae were performed by qRT-PCR. Total RNA was extracted from three early wandering staged larvae. The expression levels of transcripts were normalized with *Rp49* mRNA. The values were made from five independent experiments. Graphs represent mean ± S.D. Statistical analysis were calculated by student t-test.

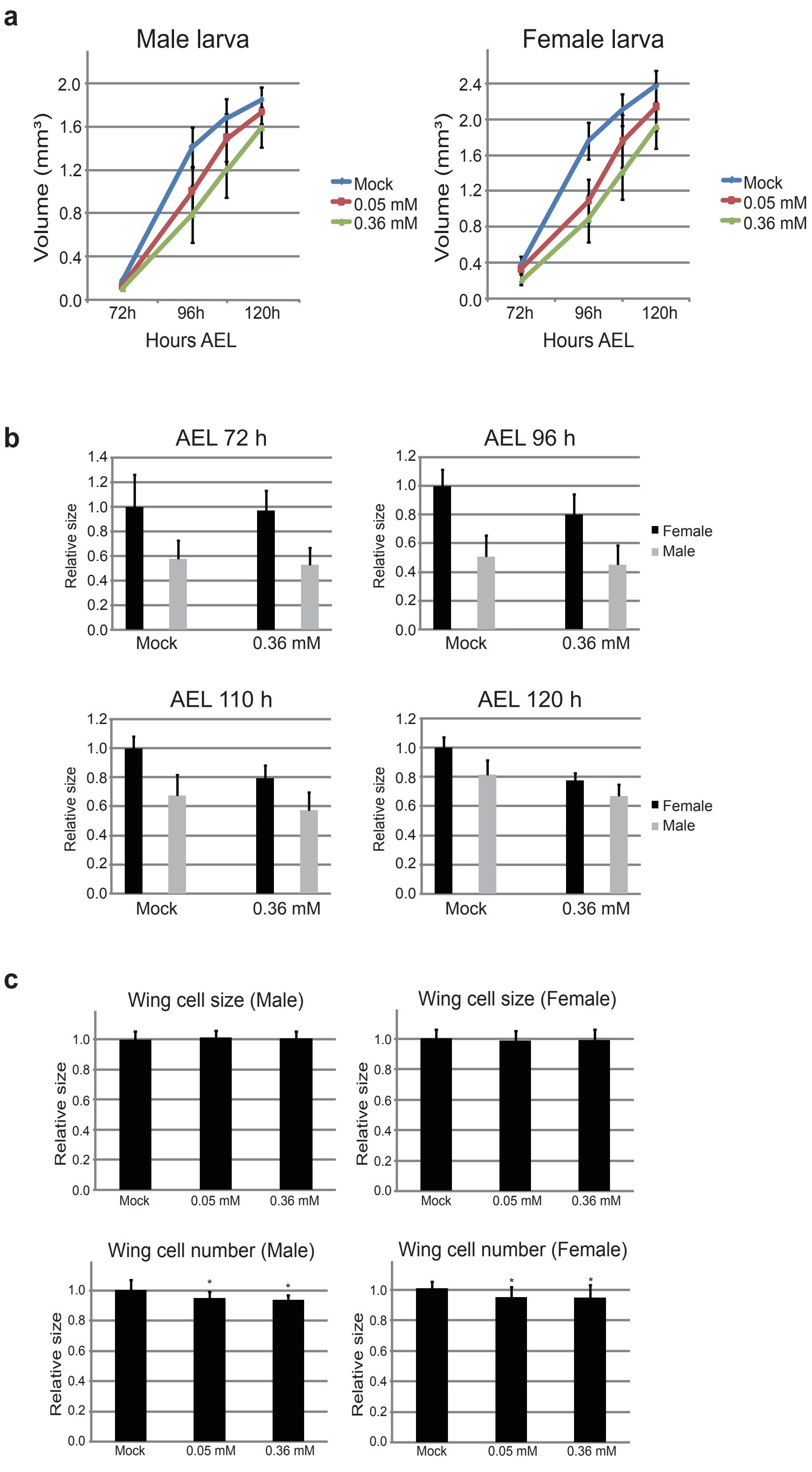

Supplementary Figure S2. Minocycline decreases larval body growth and wing cell number in both sexes. (a) Larval volumes of both male and female were measured every 24 hours from early 3rd instar larval stage (72 h AEL). ~25 larvae in each treatment were measured. (b) Larval volumes shown in (a) were re-presneted by direct comparision of female and male at each time point. (c) Wing cell size and wing cell number were measured after 5 days of eclosion. ~15 adult flies in each treatment were measured. Graphs represent mean ± S.D. *p <0.05 when compared to the respective controls (t-test).

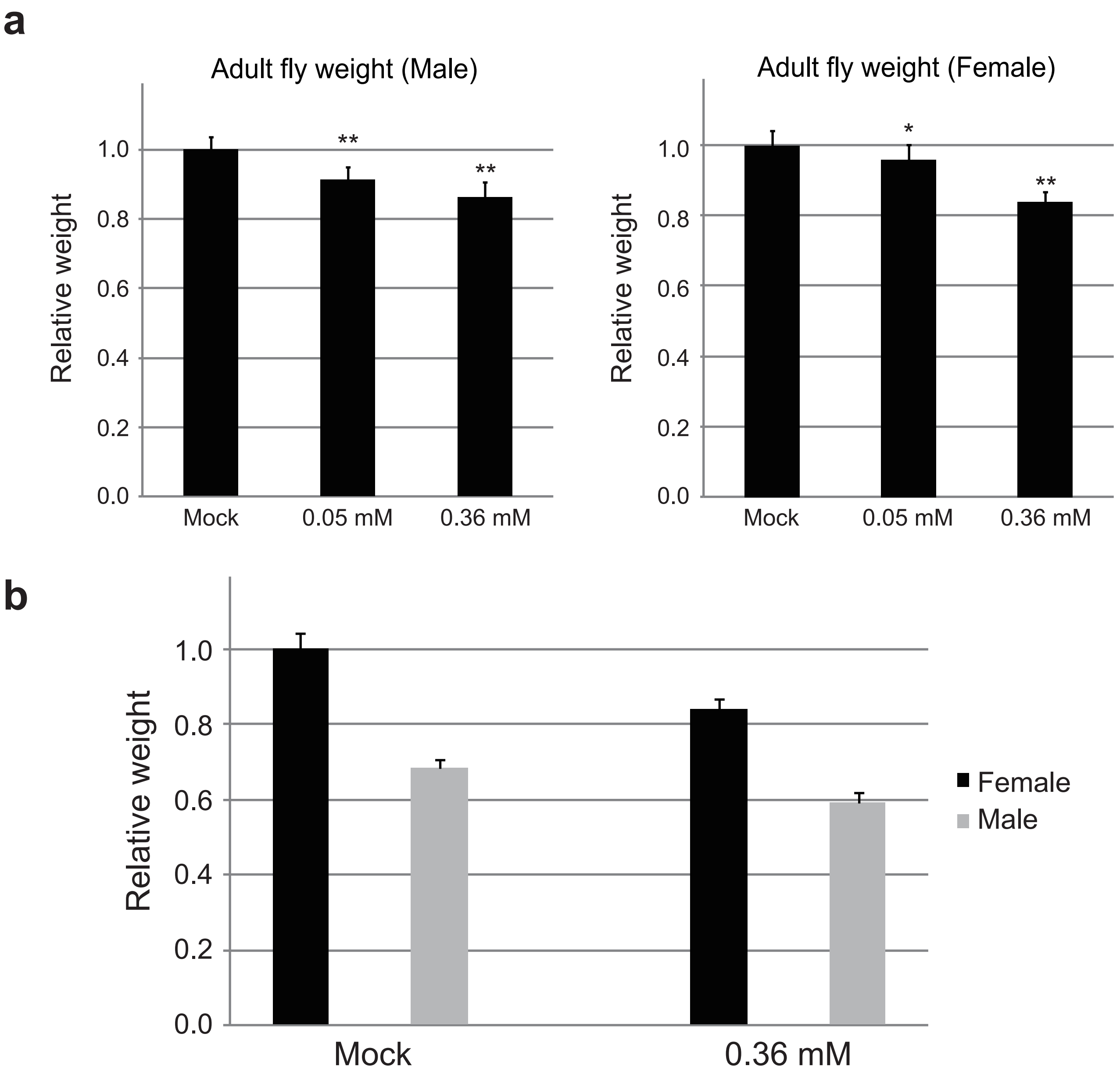

Supplementary figure S3. Minocycline treatment decreases size of five days old adult flies.
(a) Masses of five days old adult flies were measured. Feeding minocycline results in a decrease in the size of the adult flies for both sexes. ~30 flies in each treatment were measured. (b) Weights of female and male adult flies treated with 0.36mM minocycline shown in (a) were directly compared. Graphs represent mean ± S.D. *p <0.05 **p < 0.01 when compared to the respective controls (t-test).

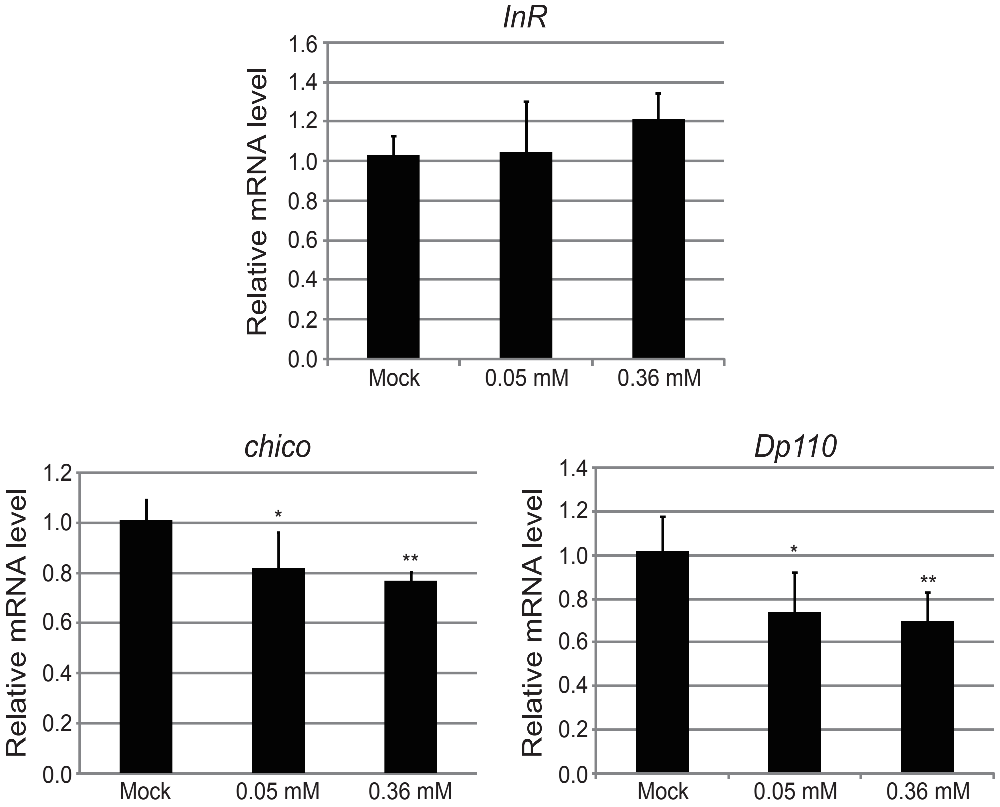

Supplementary Figure S4. Minocycline treatment suppresses the expression of genes upstream of Akt in larval tissues. Expression analysis of genes upstream of Akt [*InR* (FBgn0283499), *chico* (FBgn0024248), and *Dp110* (FBgn0015279)] were performed. Transcript levels in early 3rd instar larvae (72 h AEL) were examined by qRT-PCR. Total RNA was extracted from ten whole larval bodies. The transcript levels were normalized with *Rp49* mRNA. The values were made from five independent experiments. Graphs represent mean ± S.D. *p <0.05, **p < 0.01 when compared to the respective controls (t-test).

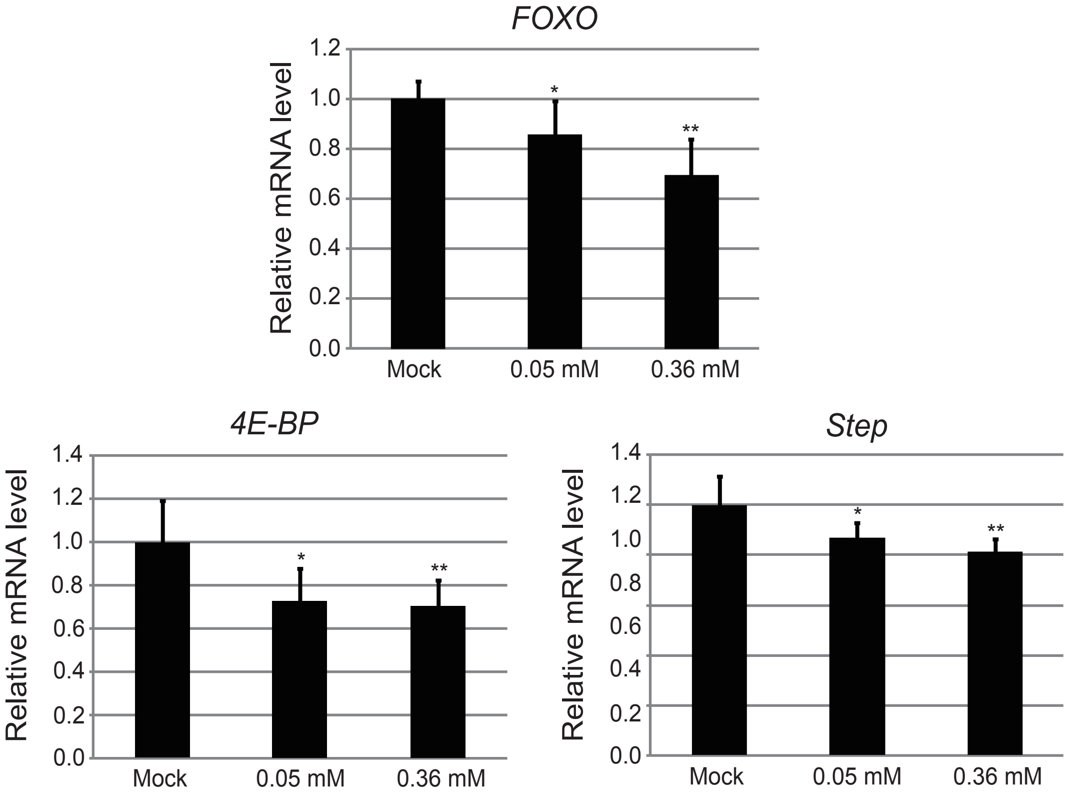

Supplementary Figure S5. Minocycline treatment suppresses expression of *FOXO* and its target genes in whole larval tissues. Transcript levels of *FOXO* (FBgn0038197) and its target genes [*4E-BP* (FBgn0261560) and *Step* (FBgn0086779)] in early 3rd instar larvae (72 h AEL) were measured by qRT-PCR. Total RNA was extracted from ten whole larval bodies. The expression levels of transcripts were normalized with *Rp49* mRNA. The values were made from five independent experiments. Graphs represent mean ± S.D. *p <0.05, **p < 0.01 when compared to the respective controls (t-test).

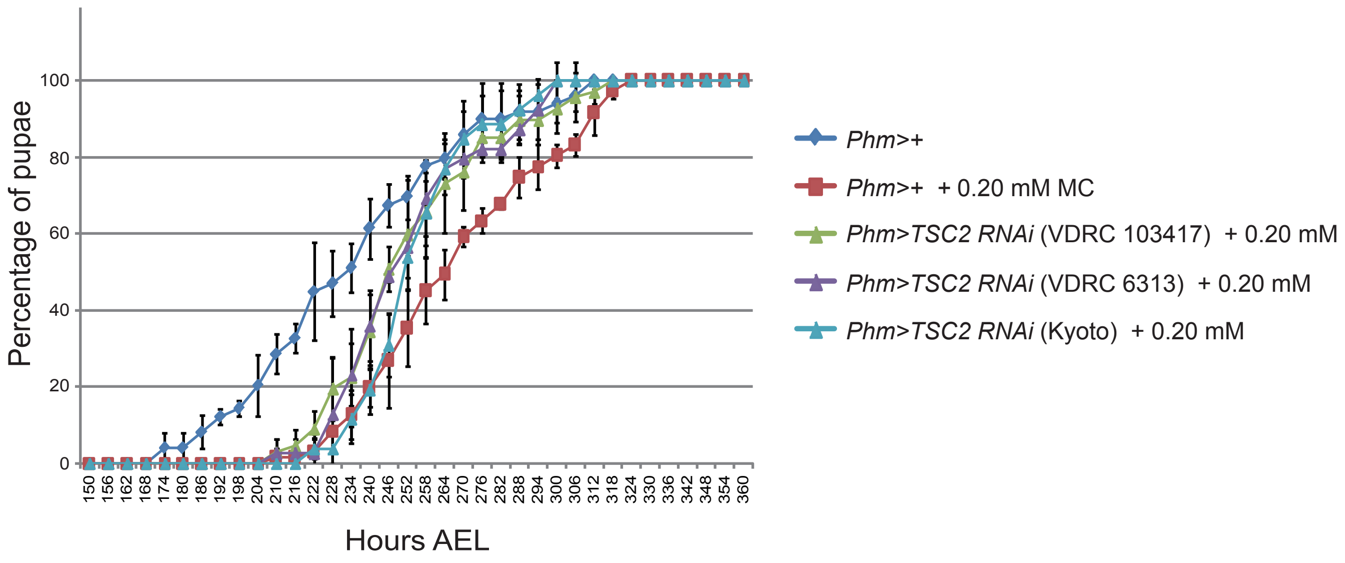

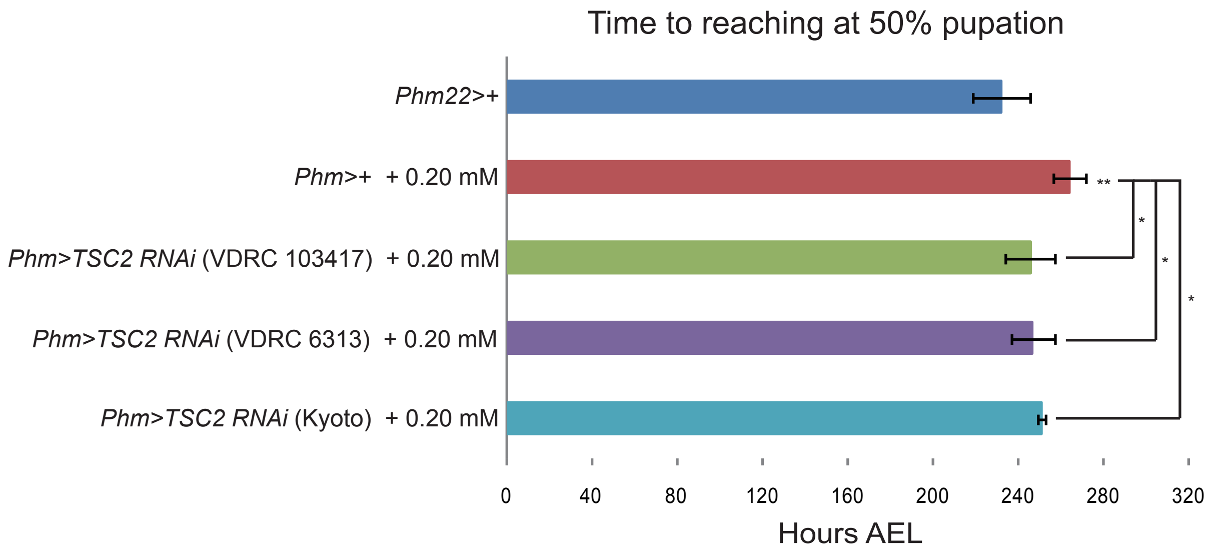

Supplementary Figure S6. TOR activation in PG using *phm Gal4* line mitigates minocycline-induced developmental delay. Enhancing TOR activity in PG using *phm Gal4* and three different TSC2 RNAi lines significantly mitigates minocylcine-induced developmental delay. RNAi line from Kyoto stock center (Kyoto) was used in Figure 4. All flies were reared on 1/4x yeast-diluted food. Three vials, each containing ~30 larvae, were examined per treatment. Graphs represent mean ± S.D. *p <0.05, **p < 0.01 when compared to the respective controls (t-test).

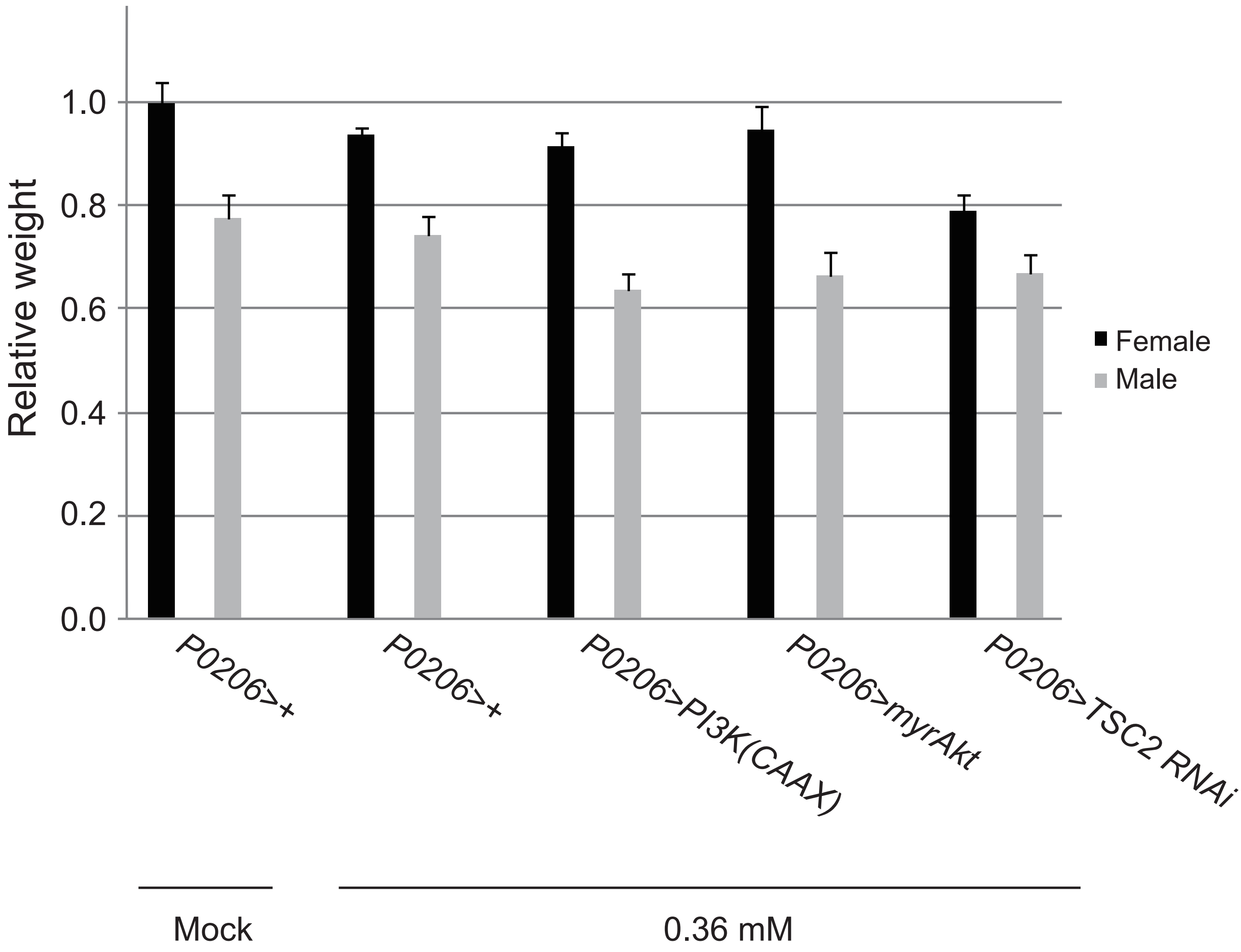

Supplementary figure S7. Comparison of female and male fly weights treated with 0.36mM minocycline. Weights of the flies treated with 0.36mM minocycline shown in c and d panels of main figure 4 were re-presented by direct comparison between female and male flies. Graphs represent mean ± S.D.

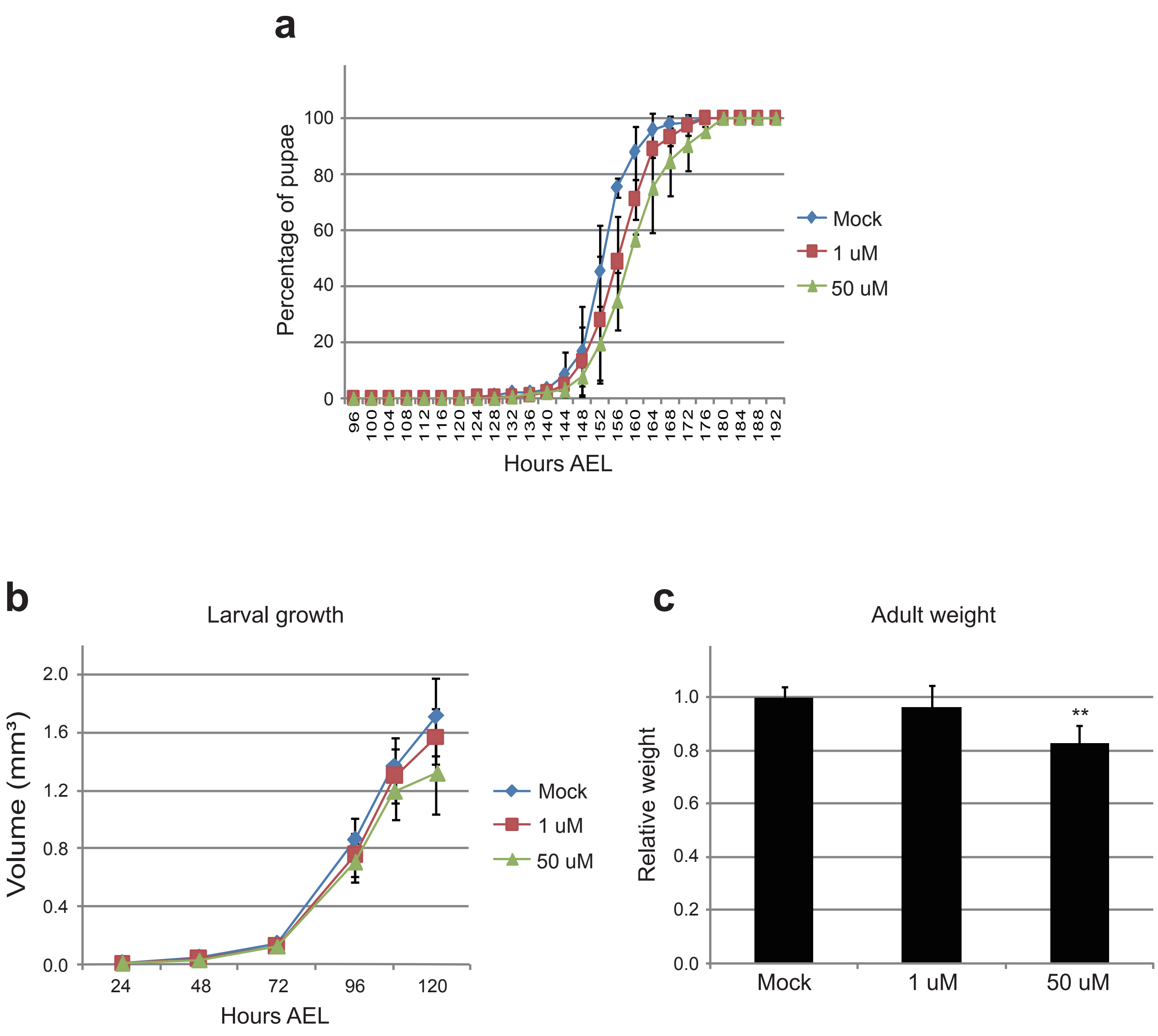

Supplementary figure S8. Rapamycin, an effective anti-tumor drug, has similar effects on larval developmen and growth as does minocycline. (a) Rapamycin treatment does-dependently delays pupation time. Three vials, each containing ~50 larvae were examined per treatment. $p=0.0045$ (1 uM) and $p<0.0001$ (50 uM) when compared to mock control (log-rank test). (b) Larval volumes were measured as the larvae developed toward puparium formation. Rapamycin treatment suppresses larval body growth in a dose-dependent manner. About 25 larvae in each treatment were measured. (c) Final size of virgin adult female was measured. Rapamycin treatment decreased final adult size. About 20 flies in each treatment were measured. Graph represents means ± S.D. $^{**}p<0.01$ when compared to the respective controls. (t-test).
